# A Dual-Functional CO_2_-Selective Membrane for Biogas Upgrading in a Microalgae Membrane Bioreactor

**DOI:** 10.3390/membranes16080279

**Published:** 2026-08-21

**Authors:** Yongze Lu, Xiaohuan Wang, Mingchao Zhu, Shouwen Chen, Zhaoxia Hu, Na Li

**Affiliations:** 1 School of Energy and Environment, Southeast University, Nanjing 210096, China; 2 Key Laboratory of Water Pollution Control and Ecological Restoration of Xizang, National Ethnic Affairs Commission, Xizang Minzu University, Xianyang 712082, China; 3 School of Engineering, Xizang Minzu University, Xianyang 712082, China; 4 Jiangsu Key Laboratory of Chemical Pollution Control and Resources Reuse, School of Environmental and Biological Engineering, Nanjing University of Science and Technology, Nanjing 210094, China

**Keywords:** microalgae adhesion, gas separation membrane, chitosan, ionic liquid, ZIF-8, mixed-matrix membrane, microalgae membrane bioreactor, biogas upgrading

## Abstract

Upgrading biogas to pipeline-quality methane requires the efficient removal of CO_2_, yet conventional physicochemical routes remain energy-intensive. Coupling a CO_2_-selective membrane with microalgal photosynthetic fixation offers a green alternative, but is constrained by the low CO_2_/CH_4_ selectivity of common membranes and the poor adhesion of microalgae to hydrophobic membrane surfaces. Here, a dual-functional composite membrane was developed that simultaneously provides CO_2_/CH_4_ sieving and a biocompatible interface for microalgal attachment, and was integrated into a microalgae membrane bioreactor (MMBR). A cellulose acetate mixed-matrix membrane incorporating polyethyleneimine-grafted ZIF-8 (CA/PZIF-8(15)) achieved a mixed-gas CO_2_ permeability of 122.3 Barrer and a CO_2_/CH_4_ selectivity of 41.17. An ionic-liquid-modified chitosan (CS/IL) coating, first optimized on a commercial flat-sheet polyethersulfone (PES) membrane used as a model surface for the adhesion study, reversed the surface charge from −30.8 to +3.75 mV, lowered the water contact angle to 51.2°, and increased the day-7 adhesion of *Scenedesmus obliquus* by ~108%. Transferring the coating onto CA/PZIF-8(15) further raised the permeability to 138 Barrer and the selectivity to 57.31, placing the composite above the 2008 Robeson upper bound. In the MMBR, CH_4_ purity reached 95.13% after 48 h; a mass balance on the recirculating gas volume indicated that essentially all of the CO_2_ removed from the gas phase permeated the membrane, of which an estimated 2% was fixed into microalgal biomass while the remainder was retained in the liquid phase. This work offers a membrane-design strategy that bridges gas-separation functionality and microalgal carbon fixation for sustainable biogas upgrading.

## 1. Introduction

Anaerobic digestion of organic waste, excess sludge, and high-strength wastewater produces biogas, a renewable energy carrier composed mainly of 50–70% CH_4_ and 30–50% CO_2_, together with trace species such as N_2_, O_2_, and H_2_S [1]. The high CO_2_ fraction lowers the calorific value of the gas and limits its use as vehicle fuel or for grid injection, so upgrading the biogas to high-purity biomethane is an essential step toward its resource utilization [2].

Conventional upgrading technologies—including water scrubbing, pressure-swing adsorption, cryogenic separation, and chemical absorption—can deliver high CH_4_ purity but generally suffer from high energy demand, high investment and operating costs, or complex operation [3,4,5]. Membrane separation, which exploits the difference in permeability of the biogas components, is attractive for its low energy consumption, simple operation, and small footprint, and is well suited to small- and medium-scale applications [6,7,8]. In parallel, the photosynthetic fixation of CO_2_ by microalgae has emerged as a green, low-carbon route that converts CO_2_ into valuable biomass [9,10,11]. Attached (biofilm) cultivation of microalgae is more efficient than suspended cultivation, avoiding the high harvesting cost that can reach 20–50% of the total cost in large-scale suspended systems [12,13,14,15].

Realizing a membrane–microalgae-coupled process on a single platform, however, faces two challenges. First, common polymeric membranes exhibit only modest CO_2_/CH_4_ selectivity, so CO_2_ cannot be transferred to the microalgal side efficiently. Mixed-matrix membranes (MMMs), in which inorganic fillers are dispersed in a polymer matrix, can improve both permeability and selectivity [16,17,18], but the choice of matrix and filler and the control of the filler–matrix interface remain decisive. Second, the surfaces of widely used membranes (e.g., polyethersulfone, polypropylene, and poly(vinylidene fluoride)) are relatively hydrophobic and carry a negative charge, which produces electrostatic repulsion against the negatively charged microalgal cells and hampers biofilm formation [19,20]. To date, membrane gas separation and microalgal adhesion have largely been studied separately, and a membrane that performs both functions simultaneously has rarely been reported [21].

The objective of this study is to develop a single flat-sheet membrane that simultaneously performs CO_2_/CH_4_ separation and supports microalgal attachment, to clarify the interfacial mechanism that governs microalgal adhesion on this membrane, and to evaluate its performance in a microalgae membrane bioreactor for biogas upgrading. To this end, a dual-functional composite membrane is designed in which a CA/PZIF-8 mixed-matrix layer performs CO_2_/CH_4_ sieving while an ionic-liquid-modified chitosan (CS/IL) coating provides a biocompatible, positively charged, hydrophilic interface for microalgal attachment. The membrane is integrated into a microalgae membrane bioreactor (MMBR) for biogas upgrading. The main contributions are: (i) a CA/PZIF-8(15) MMM with balanced permeability and selectivity that is competitive with reported MMMs under the more demanding mixed-gas conditions; (ii) a CS/IL interfacial design that, through charge reversal and enhanced hydrophilicity, markedly increases microalgal adhesion, with the mechanism resolved by surface thermodynamics and the extended DLVO (xDLVO) theory; and (iii) an integrated dual-functional membrane whose contribution to CO_2_ removal is estimated from a mass balance on the recirculating gas volume in a working MMBR.

## 2. Materials and Methods

### 2.1. Materials

Hydrophilic polyethersulfone membranes (PES, UC050; Zhongke Ruiyang Membrane Technology, Beijing, China) served as the model substrate. Cellulose acetate (acetyl 39.8%; Macklin, Shanghai, China) and poly(ether-block-amide) (Pebax MH1657; Jiabo, Foshan, China) were used as membrane matrices. Chitosan (deacetylation ≥ 90%) and epichlorohydrin were from Nanjing Jingge Chemical Technology (Nanjing, China), and the ionic liquid 1-ethyl-3-methylimidazolium acetate ([Emim][OAc], 98%) was from Shanghai Titan Scientific (Shanghai, China). Zinc nitrate hexahydrate, 2-methylimidazole, and polyethyleneimine (PEI, 30%) were used to prepare the PEI-grafted ZIF-8 filler. Melamine and ammonium chloride were used to prepare the g-C_3_N_4_/SC_3_N_4_ fillers for the Pebax comparison (Supplementary Materials). N,N-Dimethylacetamide (DMAc), methanol, ethanol, and dimethyl sulfoxide (DMSO, Sinopharm Chemical Reagent, Shanghai, China) were used as solvents. *Scenedesmus obliquus* (FACHB-416; Institute of Hydrobiology, Chinese Academy of Sciences, Wuhan, China) was cultured in BG11 medium. Other reagents were of analytical grade.

### 2.2. Preparation of the PZIF-8 Filler

ZIF-8 was synthesized at room temperature. Briefly, 0.736 g Zn(NO_3_)_2_·6H_2_O and 1.624 g 2-methylimidazole were dissolved in methanol (50 mL in total); the two solutions were combined and stirred for 4 h. The resulting suspension was centrifuged, washed with deionized water, and dried at 80 °C for 12 h to obtain ZIF-8 powder [22]. The PEI-grafted ZIF-8 (PZIF-8) was prepared by the same procedure, except that 0.18 g polyethyleneimine was first dissolved in 20 mL methanol and combined with 30 mL methanol containing 1.624 g 2-methylimidazole to form a PEI–Hmim solution before adding the zinc nitrate solution, so that PEI was incorporated in situ during framework formation [23].

The g-C_3_N_4_ and SC_3_N_4_ fillers used for the Pebax-based comparison membranes were prepared as follows. Graphitic carbon nitride (g-C_3_N_4_) nanosheets were synthesized by thermal polymerization: melamine (2.0 g) and NH_4_Cl (4.0 g) were ground together, transferred to a ceramic crucible, and heated in a muffle furnace to 550 °C at 5 °C min^−1^ and held for 4 h, giving a pale-yellow solid (yield ~45.8%), which was ground into powder. Sulfonation was carried out following Lu et al. [24]: 1 g of the g-C_3_N_4_ powder was dispersed in 20 mL DMSO, and 0.5 mL 1,4-butane sultone and 0.2 g NaOH were added; the mixture was stirred at 105 °C for 6 h under N_2_. The product (denoted SC_3_N_4_) was washed several times with ethanol, collected by centrifugation, and dried under vacuum at 80 °C.

### 2.3. Preparation of the CA-Based Mixed-Matrix Membranes

All membranes in this study were prepared as flat-sheet (film) membranes by solution casting. The CA-based MMMs are the membranes used throughout this study; the Pebax-based membranes were prepared for comparison, and their characterization is reported in the Supplementary Materials.

For the CA-based MMMs, 1 g of CA was dispersed in 5 mL of N,N-dimethylacetamide (DMAc) to give a homogeneous suspension, and a pre-calculated amount of ZIF-8 or PZIF-8 was separately dispersed in another 5 mL of DMAc. The two suspensions were then combined, stirred, and left to stand for 12 h. The mixed suspension was cast onto a glass plate and spread with a doctor blade, and the film was dried at 60 °C for 12 h. Membranes with filler loadings of 10, 15, and 20 wt% were denoted as CA/ZIF-8(x) and CA/PZIF-8(x), where the filler loading (wt%) is defined as m_filler/(m_filler + m_polymer). The solid-to-liquid ratio of the casting solutions was kept constant at 1 g of CA per 10 mL of DMAc for all compositions.

**Figure 1:**
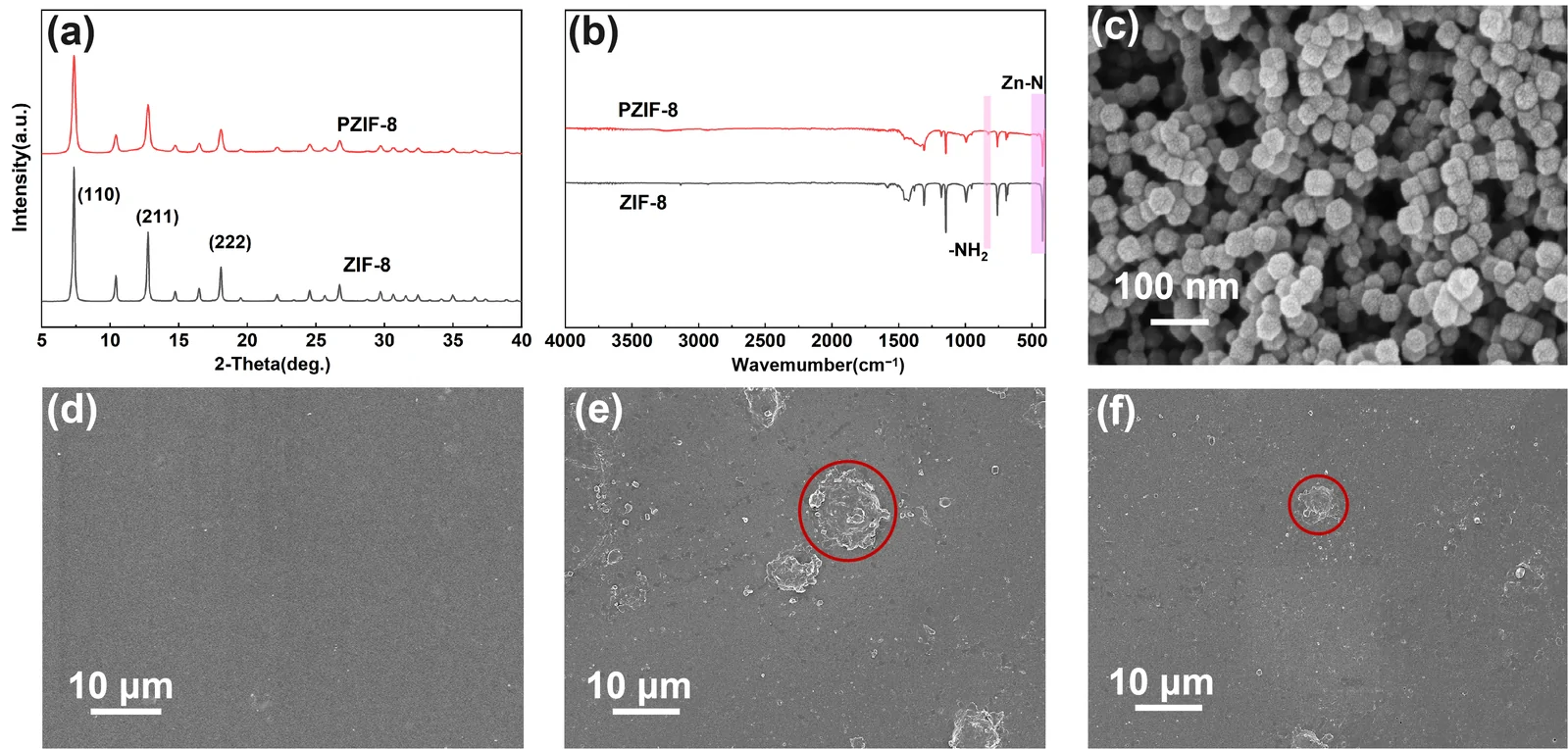
Characterization of the PZIF-8 filler and the CA-based mixed-matrix membranes. (**a**) XRD patterns and (**b**) FTIR spectra of ZIF-8 and PZIF-8; (**c**) SEM image of PZIF-8; surface SEM images of (**d**) pristine CA, (**e**) CA/ZIF-8(15) and (**f**) CA/PZIF-8(15). The red circles show the filler particles on the membrane surface. They indicate that the particle aggregation and size of PZIF-8 in figure (**f**) are reduced compared to ZIF-8 in figure (**e**).

The Pebax-based MMMs were also prepared by solution casting. Pebax (0.8 g) was dissolved in 15.2 g of ethanol/water (7:3, *v*/*v*) under reflux at 80 °C for 2 h to give a 5 wt% solution. C_3_N_4_ or SC_3_N_4_ was dispersed in the same solvent mixture by five successive 30 min ultrasonication steps, added to the Pebax solution, and refluxed at 80 °C for a further 12 h. After ultrasonic degassing, the casting solution was poured into a glass mould and dried at 40 °C for 24 h, giving filler loadings of 10, 20 and 30 wt% (Figures S1–S3). Pristine Pebax membranes were prepared by the same procedure without filler. The membrane codes and compositions are summarized in Table S1. The morphology of the membranes is shown in Figure 1d–f and Figure S2 and Figure S6.

### 2.4. Preparation of the CS/IL Coating and the Composite Membrane

Chitosan (CS) solutions of 1.0, 1.5, 1.8, and 2.0 wt% were prepared by dissolving chitosan in 1% acetic acid under stirring for 6 h, followed by ultrasonication to remove bubbles. To graft the ionic liquid, 0.1 g CS was dissolved in 10 mL of 1% acetic acid; 100 µL epichlorohydrin (epichlorohydrin/CS molar ratio 2:1) was added with a base catalyst to neutralize the HCl released, and the system was heated to 40 °C and stirred for 8 h. [Emim][OAc] was then added, and the mixture was stirred at 60 °C for 12–24 h to obtain the IL-grafted chitosan (CS/IL) solution [25]. The IL/CS mass ratios were 30%, 50%, and 100%. The CS and CS/IL solutions were spin-coated onto the PES substrate and vacuum-dried for 12 h, giving the coated membranes PES-x%CS and PES-x%CS/IL. Here, the commercial flat-sheet PES membrane served only as a model substrate. It provides a smooth, reproducible, and readily available surface on which the coating composition could be screened and the interfacial properties measured, so that the design of the adhesion interface was decoupled from the gas-separation layer. The dual-functional composite membrane was prepared by spin-coating the optimized CS/IL solution onto a CA/PZIF-8(15) membrane and drying at 60 °C for 24 h, yielding IL-CS@CA/PZIF-8(15).

### 2.5. Characterization

The chemical structure, morphology, crystallinity, and thermal and mechanical properties of the fillers and membranes were characterized by Fourier-transform infrared spectroscopy (FTIR; Nexus 670, Nicolet, Madison, WI, USA), field-emission scanning electron microscopy (SEM; Regulus 8100, Hitachi High-Tech, Tokyo, Japan), X-ray diffraction (XRD), N_2_ adsorption–desorption, and tensile testing. Surface wettability was evaluated by water contact angle measurements (OCA 20, Dataphysics, Filderstadt, Germany), and surface charge was determined using a solid-surface Zeta potential analyzer for the membranes (SurPASS 3, Anton Paar, Graz, Austria) and a nanoparticle Zeta potential analyzer for the microalgal cells (Zetasizer ZS90, Malvern, UK). The optical density of the microalgal suspensions was measured with a UV–vis spectrophotometer (UV2300, Tianmei, Shanghai, China).

### 2.6. Interfacial Free Energy and xDLVO Analysis

Contact angles of water, diiodomethane, and ethylene glycol on the membranes and on the *S. obliquus* cell layer were measured to obtain the surface free energy components (total, Lifshitz–van der Waals, and acid–base; electron-acceptor and electron-donor) [26]. The adhesion free energy (ΔG_adh_) between the membrane and the microalgae was calculated from surface thermodynamics, and the total interaction energy as a function of separation distance was evaluated using the extended DLVO (xDLVO) theory [27], which combines Lifshitz–van der Waals, electrostatic, and acid–base interactions [28]. The corresponding equations are given in Text S1 of the Supplementary Materials.

### 2.7. Gas Separation and Microalgal Adhesion Tests

The CO_2_/CH_4_ separation performance was measured with a CO_2_/CH_4_ (50/50, *v*/*v*) mixed gas at 25 °C and 2 bar, and the CO_2_ permeability (Barrer) and CO_2_/CH_4_ selectivity were calculated. For the adhesion tests, activated *S. obliquus* suspension was diluted with BG11 to OD_680_ = 0.6 and adjusted to pH 7. Membrane coupons were placed in six-well plates, 5 mL of algal suspension was added to each well, and 100 µL of BG11 was supplied daily. Samples were taken on days 1, 3, 5, and 7; the attached cells were resuspended by rinsing and sonication, quantified by OD_680_, and converted to areal biomass (g·m^−2^) using a dry-cell-weight calibration curve.

### 2.8. Construction and Operation of the MMBR

The lab-scale MMBR consisted of a gas chamber (effective volume of 136 mL, height of 33.5 mm) and a liquid chamber (effective volume of 92 mL, height of 22.5 mm) separated by the composite membrane, which was mounted as a flat-sheet disc between the two chambers with an effective membrane area of 40.7 cm^2^. The reactor was equipped with LED illumination and a recirculating BG11 liquid phase (Figure S11). In each run, the reactor was charged with simulated biogas (CO_2_/CH_4_ = 50/50, *v*/*v*), which was then recirculated through the module in a closed loop at a controlled flow rate of 100 mL·min^−1^ and a constant gauge pressure of 2 bar (3 bar absolute); the liquid phase (BG11) was recirculated at 20 mL·min^−1^. The permeate-side headspace (gas outlet 2) was sampled at fixed daily intervals to monitor the CH_4_ purity. The effects of ambient temperature (15, 20, 25, 30 °C at 5000 lx) and light intensity (5000, 7500, 10,000, 12,500 lx at 25 °C) were investigated, and the long-term performance was monitored over seven days.

## 3. Results and Discussion

### 3.1. Characterization of the Filler and the CA-Based Membranes

The three components of the membrane were selected to meet the two functions required of it. Cellulose acetate was chosen as the matrix because it is a low-cost, commercially available polymer with good film-forming ability and a well-documented intrinsic CO_2_/CH_4_ selectivity, and because its hydrophilic and biocompatible surface is favourable for the subsequent microalgal attachment. ZIF-8 was chosen as the filler because its sodalite cages provide an effective aperture that lies between the kinetic diameters of CO_2_ and CH_4_, and because of its high surface area and its chemical and thermal stability. Polyethyleneimine was used to graft the ZIF-8 surface in order to address the interfacial voids that commonly form between a rigid filler and a polymer matrix, and to introduce amine groups with a high affinity for CO_2_.

The PEI grafting of ZIF-8 and the formation of the CA-based MMMs were confirmed by XRD, FTIR, and SEM (Figure 1). The XRD patterns retained the characteristic ZIF-8 reflections after PEI grafting, and the FTIR spectra showed the additional bands expected from the grafted PEI, indicating that the crystalline framework was preserved while amine functionality was introduced (Figure 1a,b). The SEM image of PZIF-8 showed uniform, well-defined nanoparticles (Figure 1c). Surface SEM images of pristine CA, CA/ZIF-8(15), and CA/PZIF-8(15) (Figure 1d–f) showed that the fillers were dispersed in the CA matrix with only minor local aggregation [18]. Because a cross-sectional SEM was not obtained, the filler dispersion is inferred here from the surface morphology together with the XRD and FTIR evidence; a cross-sectional analysis with EDS mapping is left for future work. The CA/PZIF-8(15) membrane exhibited a tensile strength of 26.1 MPa and an elongation at break of 9.8% (Figure S4b), indicating that PZIF-8 incorporation did not compromise the mechanical integrity of the matrix.

### 3.2. CO_2_/CH_4_ Separation Performance

**Figure 2:**
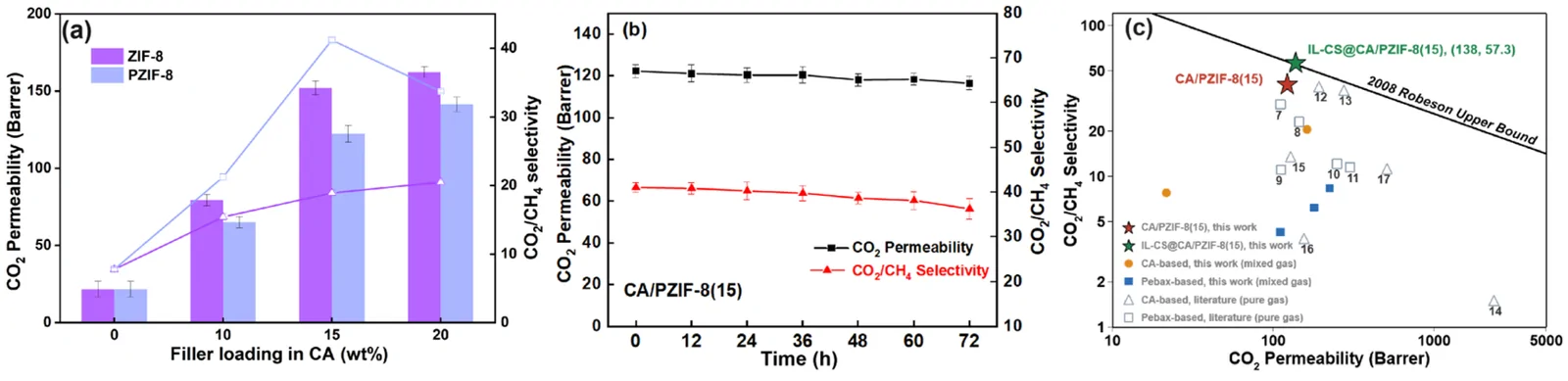
Gas separation performance of the CA-based mixed-matrix membranes. (**a**) Effect of PZIF-8 loading on CO_2_ permeability and CO_2_/CH_4_ selectivity, the bar chart represents CO_2_ permeability and the line graph shows CO_2_/CH_4_ selectivity.; (**b**) long-term stability of CA/PZIF-8(15) at 2 bar and 25 °C; (**c**) comparison with the 2008 Robeson upper bound and with the literature mixed-matrix membranes (numbered 1–11 as in Table 1). Filled symbols: this work (mixed gas); open symbols: the literature (pure gas unless otherwise noted in Table 1).

The effect of PZIF-8 loading on the separation performance of the CA-based MMMs is shown in Figure 2a. Both the CO_2_ permeability and the CO_2_/CH_4_ selectivity increased with loading up to 15 wt%, reaching a selectivity of 41.17; at 20 wt%, the selectivity dropped to 33.8, indicating that excessive filler causes particle aggregation and the formation of non-selective voids [29].

The improvement in CO_2_/CH_4_ selectivity originates from two complementary contributions. First, the sodalite cages of ZIF-8 have an effective aperture that lies between the kinetic diameters of CO_2_ (0.33 nm) and CH_4_ (0.38 nm), which introduces a size-sieving pathway favouring CO_2_ diffusion. Second, the amine groups introduced by PEI grafting are CO_2_-philic and increase the sorption selectivity towards CO_2_ through acid–base interaction. In addition, the grafted PEI chains fill the interfacial voids between the filler and the CA matrix, suppressing the non-selective voids that commonly limit the selectivity of mixed-matrix membranes. The interaction between the filler surface and the CA chains also restricts the local segmental mobility of the polymer in the interfacial region, which further favours the smaller CO_2_ molecule.

The CA/PZIF-8(15) membrane maintained a relatively stable permeability and selectivity over 72 h (Figure 2b). As shown in the Robeson diagram (Figure 2c), the CA/PZIF-8(15) point (122.3 Barrer, 41.17) lies below the 2008 upper bound for CO_2_/CH_4_ [30,31]; however, this performance was obtained under mixed-gas conditions, whereas most of the literature values in Table 1 were measured with pure gases. Because competitive sorption and plasticization generally lower the mixed-gas selectivity, the present membrane is in fact competitive with pure-gas MMMs of comparable permeability.

**Table 1:** Comparison of the CO_2_/CH_4_ separation performance of the CA/PZIF-8 membrane with mixed-matrix membranes reported in the literature.

No.	Membrane	CO_2_ Permeability (Barrer)	CO_2_/CH_4_ Selectivity	Test Gas	T (°C)	P (Bar)	Ref.
1	Pristine Pebax	110.67	4.27	CO_2_/CH_4_ (50/50 vol%)	25	2	This work
2	Pebax/C_3_N_4_(30)	179.67	6.2	CO_2_/CH_4_ (50/50 vol%)	25	2	This work
3	Pebax/SC_3_N_4_(30)	224	8.33	CO_2_/CH_4_ (50/50 vol%)	25	2	This work
4	Pristine CA	21.67	7.77	CO_2_/CH_4_ (50/50 vol%)	25	2	This work
5	CA/ZIF-8(20)	162.33	20.47	CO_2_/CH_4_ (50/50 vol%)	25	2	This work
6	CA/PZIF-8(15)	122.33	41.17	CO_2_/CH_4_ (50/50 vol%)	25	2	This work
7	Pebax/ZIF-7 (22 wt%)	111	30	Pure gas	25	3.75	[32]
8	Pebax/ZIF-7 (8 wt%)	145	23	Pure gas	25	3.75	[32]
9	Pebax/PSF	111.6	11.05	CO_2_/CH_4_ mixed gas	30	6	[33]
10	Pebax-30%MIL-53/PSF	249.9	12.1	CO_2_/CH_4_ mixed gas	30	6	[33]
11	Pebax-30%NH_2_-MIL-53/PSF	300.8	11.5	CO_2_/CH_4_ mixed gas	30	6	[33]
12	U-150@CA	192.8	39.3	Pure gas	25	3	[34]
13	U-200@CA	275.3	37.2	Pure gas	25	3	[34]
14	0.2His_ZIF/CA	2360	1.5	Pure gas	25	3	[35]
15	1.0His_ZIF/CA	128.4	13.49	Pure gas	25	3	[35]
16	3_MF/CA	155.56	3.87	Pure gas	25	3	[36]
17	3.0AP_3_MF/CA	510.64	11.23	Pure gas	25	3	[36]

Notes: Numbers 1–11 correspond to the literature points labelled in Figure 2c. Membranes in this work were tested with a CO_2_/CH_4_ (50/50 vol%) mixed gas, whereas the literature values were measured with pure gases; mixed-gas selectivity is generally lower owing to competitive sorption and plasticization. 1 Barrer = 10^−10^ cm^3^(STP)·cm·cm^−2^·s^−1^·cmHg^−1^.

### 3.3. Interfacial Design and Microalgal Adhesion

**Figure 3:**
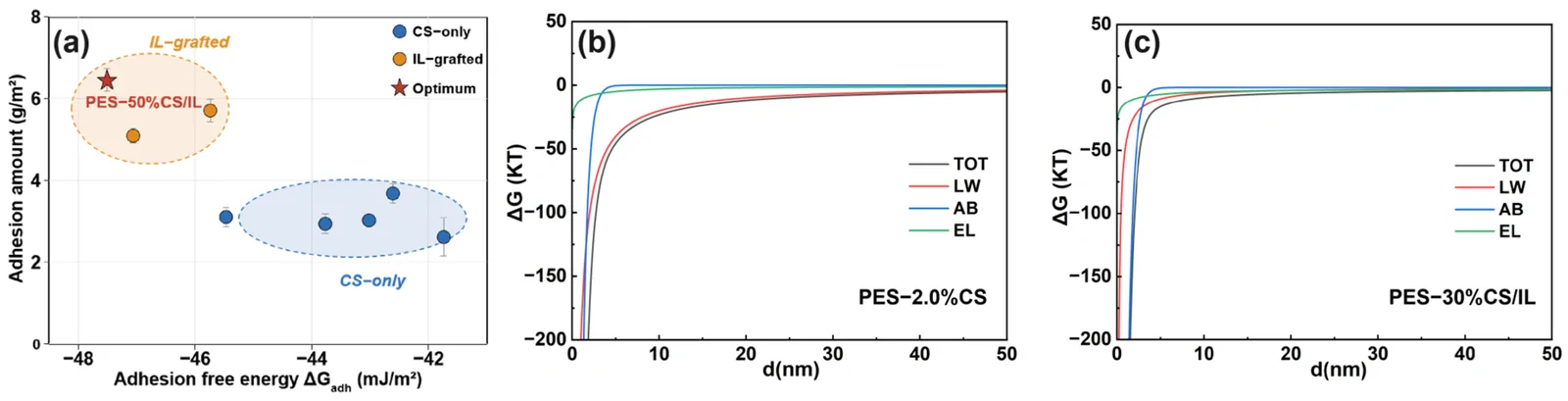
Interfacial mechanism of microalgal adhesion. (**a**) Correlation between the adhesion free energy ΔG_adh_ and the day-7 adhesion amount, showing distinct CS-only and IL-grafted clusters; interaction-energy profiles versus separation distance for (**b**) PES-2.0%CS and (**c**) PES-50%CS/IL. The coating was optimized on a porous PES model substrate.

The interfacial properties of the CS/IL coating and their effect on microalgal adhesion are summarized in Table 2 and Figure 3. Coating the PES model substrate with chitosan raised the water contact angle (e.g., 86.6° for PES-2.0%CS), whereas grafting the chitosan with the ionic liquid markedly lowered it to 51.2° for PES-50%CS/IL, reflecting a substantial increase in hydrophilicity. More importantly, the surface Zeta potential reversed from −30.8 mV for pristine PES to positive values after coating (+3.75 mV for PES-50%CS/IL), switching the interaction with the negatively charged *S. obliquus* (−39.73 mV) from repulsion to attraction [19]. Consistently, the adhesion free energy became more negative, from −41.74 mJ m^−2^ for PES to −47.51 mJ m^−2^ for PES-50%CS/IL, with the acid–base component dominating the change.

Figure 3a correlates the adhesion free energy with the measured day-7 adhesion amount. The samples segregate into two clusters: CS-only coatings yield only a modest adhesion gain, whereas IL-grafted coatings shift all samples to markedly more negative ΔG_adh_ and substantially higher adhesion. This indicates that the enhanced adhesion is governed primarily by the interfacial free energy established through IL grafting rather than by chitosan concentration or wettability alone [37]. The xDLVO interaction-energy profiles of the two representative membranes (Figure 3b,c) support this picture: for PES-2.0%CS, the potential well is shallow (about −150 kT), whereas for PES-50%CS/IL, the well is much deeper (down to about −600 kT), with the acid–base interaction becoming dominant within ~5 nm and the electrostatic interaction turning from repulsive to attractive over 5–20 nm. Accordingly, the day-7 adhesion on PES-50%CS/IL reached 6.47 g m^−2^, ~108% higher than on PES-2.0%CS (3.11 g m^−2^) [38]. The coating was optimized on PES as a model substrate to decouple the interface design from the gas-separation layer.

### 3.4. Dual-Functional Composite Membrane and the MMBR

Coating the optimized CS/IL layer onto CA/PZIF-8(15) improved both transport metrics simultaneously: the CO_2_ permeability rose from 122.3 to 138 Barrer, and the mixed-gas CO_2_/CH_4_ selectivity increased from 41.17 to 57.31 (Figure 2c). Such a simultaneous enhancement is uncommon, because polymer coatings usually trade permeability for selectivity. Here, the CO_2_-philic imidazolium acetate moieties of the CS/IL layer provide a facilitated-transport pathway that preferentially accelerates CO_2_ while adding resistance to the non-polar CH_4_ [39]. The fact that the selectivity increased rather than deteriorated also indicates that a continuous, defect-free CS/IL layer was successfully established on the mixed-matrix substrate, confirming the transfer of the coating chemistry from the PES model substrate to the gas-separation membrane. As a result, the composite crosses the 2008 Robeson upper bound, whereas the pristine CA/PZIF-8(15) lies below it (Figure 2c).

**Figure 4:**
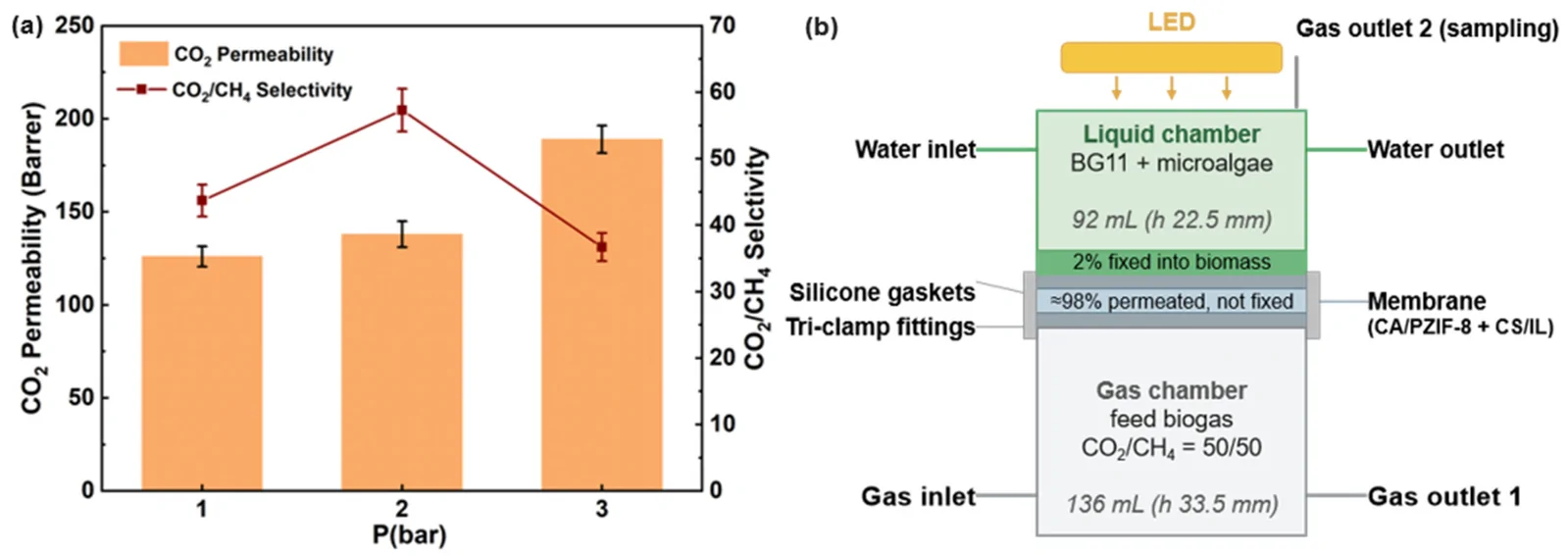
The dual-functional composite membrane in the microalgae membrane bioreactor. (**a**) Effect of transmembrane pressure on the separation performance of IL-CS@CA/PZIF-8(15) at 25 °C; (**b**) cross-section of the lab-scale MMBR module and the two CO_2_ removal pathways. Essentially all of the CO_2_ removed from the gas phase permeates the membrane; the percentages indicate the estimated fraction that is fixed into microalgal biomass and the fraction that remains unfixed in the liquid phase.

The composite membrane was assembled into the MMBR (Figure 4). The transmembrane pressure influenced the separation performance of IL-CS@CA/PZIF-8(15), with 2 bar providing the best balance between CO_2_ flux and selectivity (Figure 4a); at 3 bar, the selectivity decreased, consistent with more non-selective permeation through interfacial defects under a higher driving force. In the module (Figure 4b), the feed biogas is supplied to the gas chamber, CO_2_ permeates selectively through the composite membrane, and the attached microalgae on the permeate side fix part of the permeated CO_2_ under illumination, so that the two functions act in synergy.

### 3.5. Effect of Operating Conditions

**Figure 5:**
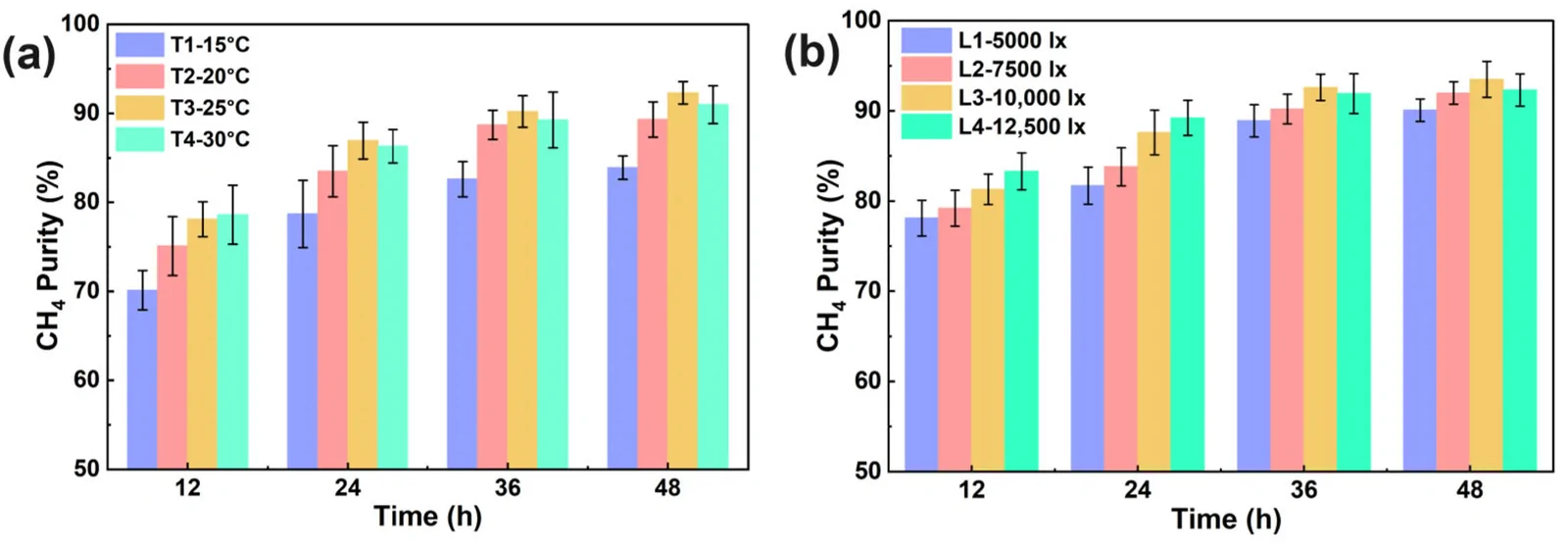
Effect of operating conditions on biogas upgrading in the microalgae membrane bioreactor. CH_4_ purity over 48 h at (**a**) different ambient temperatures (15, 20, 25 and 30 °C) under 5000 lx and (**b**) different light intensities (5000, 7500, 10,000 and 12,500 lx) at 25 °C.

The effects of ambient temperature and light intensity on biogas upgrading are shown in Figure 5. Within the tested range, both parameters influenced the CH_4_ purity attained over 48 h, mainly through their effect on the membrane permeation behaviour and, secondarily, on the photosynthetic activity of the attached microalgae. The best performance was obtained at 25 °C and 10,000 lx, which were therefore adopted as the operating conditions for the subsequent long-term tests. At a fixed pressure of 2 bar, the CH_4_ purity increased with reaction time under all conditions and rose with temperature up to an optimum at 25 °C, where it reached 91.71% at 48 h (Figure 5a); the initial purity (60.9–66.9%) already exceeded the 50% feed value, confirming that the composite membrane provides the initial separation. A higher temperature enhances gas diffusion within the membrane and thus the CO_2_ permeation flux, whereas the slightly lower purity at 30 °C reflects partial photoinhibition of the microalgae and a minor plasticization of the matrix [40]. At 25 °C, the CH_4_ purity first increased and then decreased with light intensity, peaking at 95.13% at 48 h under 10,000 lx and declining at 12,500 lx owing to photoinhibition (Figure 5b) [41]. Accordingly, 25 °C and 10,000 lx were adopted for the subsequent long-term tests.

### 3.6. Long-Term Operation, Mass Balance and Techno-Economics

**Figure 6:**
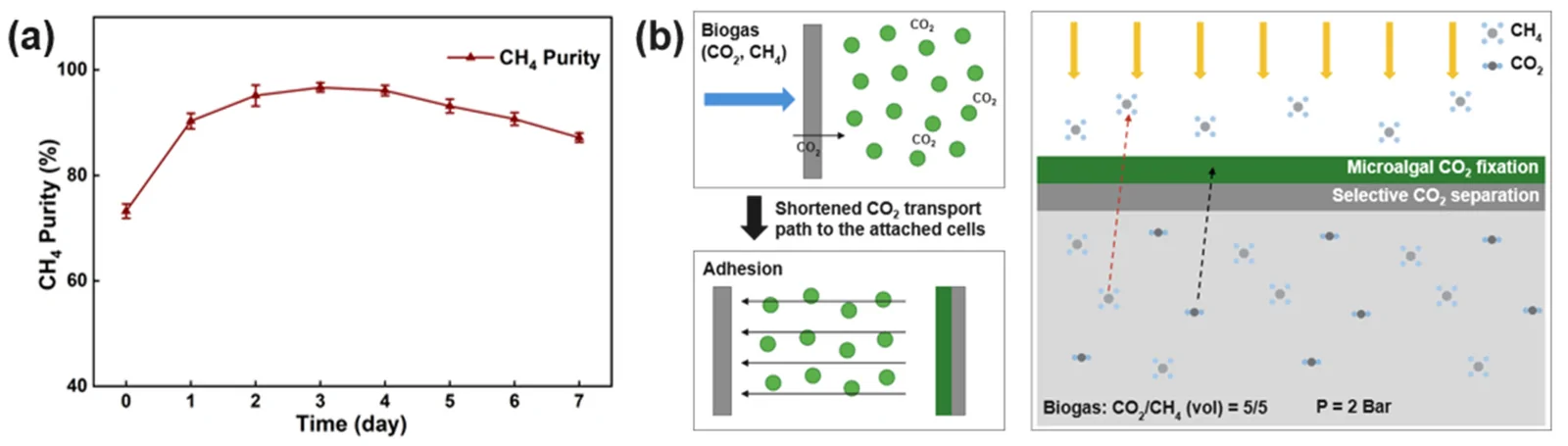
Long-term performance and the underlying interfacial mechanism. (**a**) CH_4_ purity of the microalgae membrane bioreactor over seven days of continuous operation at 25 °C and 10,000 lx; (**b**) schematic of the interfacial mass-transfer and conversion mechanism. Arrows show the direction of gas movement.

Under the optimized conditions, the CH_4_ purity rose quickly during the first two days, reached 96.67% on day 3, and then declined to 87.16% by day 7 (Figure 6a). The decline is attributed to biofilm ageing and the associated increase in mass-transfer resistance: as the biofilm thickens, light penetration and CO_2_ transport to the inner cells are progressively limited, lowering the fixation rate, while ageing cells may release extracellular polymeric substances that locally hinder transport [42]. The interfacial mass-transfer mechanism, in which the membrane acts as a directional carbon-source supply to the attached biofilm, is illustrated in Figure 6b. Adhesion shortens the transport path between the membrane and the cells relative to suspended cultivation, whereas the biofilm itself adds mass-transfer resistance as it thickens.

The relative contributions of the two removal pathways were assessed by a mass balance on the 136 mL gas headspace, which was charged once and then recirculated in a closed loop (Section 2.8). Taking CH_4_ as conserved and treating the gas as ideal at the operating temperature and pressure (25 °C, 3 bar absolute), the CH_4_ purity of 95.13% reached at 48 h corresponds to a total CO_2_ removal of approximately 344 mg from the gas phase. Because the gas loop is closed, the membrane is the only route by which CO_2_ can leave the gas chamber, so that essentially all of this CO_2_ left the gas phase by permeation through the composite membrane.

The fraction of the permeated CO_2_ that was converted into biomass was estimated independently from the areal biomass increment measured over the corresponding 48 h period. Between day 1 and day 3, the areal biomass on the coated membrane increased from 2.38 g m^−2^ on day 1 to 3.35 g m^−2^ (Figure S9), which, taking a cellular carbon content of 48% and a CO_2_ /C mass ratio of 44/12, corresponds to an areal fixation rate of ≈0.86 g CO_2_ m^−2^·d^−1^ [43]. Over the effective membrane area of 40.7 cm^2^, this amounts to ≈7.0 mg CO_2_ in 48 h, which is about 2% of the CO_2_ removal from the gas phase.

The remaining ≈98% permeated the membrane but was not fixed into biomass. Under the present conditions, this fraction is expected to reside mainly in the recirculating liquid phase as dissolved CO_2_ and bicarbonate. A Henry’s law estimate for the 92 mL liquid volume at the final gas composition gives ≈20 mg of dissolved CO_2_, and the associated bicarbonate pool is larger and strongly pH-dependent, so the dissolved inorganic carbon reservoir is of the same order as the total CO_2_ removed from the gas phase. This reservoir was not determined experimentally, so the microalgal share is reported here as an estimate; it further assumes that the gas loop is free of leakage and that the growth rate measured in the adhesion tests is representative of the biofilm in the reactor.

Membrane permeation therefore governs the upgrading process, while the attached microalgae act as a comparatively small but continuous sink that consumes permeate-side CO_2_ and helps to sustain the transmembrane concentration gradient, giving a “separation-plus-intensification” synergy [21]. A qualitative comparison with representative biogas upgrading technologies is given in Table 3 [5,44].

## 4. Conclusions

A dual-functional composite membrane was developed that combines a CA/PZIF-8 mixed-matrix layer for CO_2_/CH_4_ sieving with an ionic-liquid-modified chitosan coating that provides a biocompatible interface for microalgal attachment. The CA/PZIF-8(15) membrane delivered a mixed-gas CO_2_ permeability of 122.3 Barrer and a CO_2_/CH_4_ selectivity of 41.17. The CS/IL coating reversed the surface charge and enhanced hydrophilicity, increasing the day-7 adhesion of *S. obliquus* by ~108%, with the interfacial free energy—dominated by the acid–base component—identified as the controlling factor. Transferring the coating onto CA/PZIF-8(15) raised the permeability and selectivity to 138 Barrer and 57.31, respectively, placing the composite above the 2008 Robeson upper bound. In the MMBR, the CH_4_ purity reached 95.13% at 48 h, with membrane permeation governing the removal and an estimated 2% of the CO_2_ removed from the gas phase being fixed into microalgal biomass. The main remaining challenge is the long-term stability of the biofilm; future work will address biofilm renewal and light-path optimization to mitigate the performance decline. Overall, the dual-functional membrane offers a viable strategy that bridges gas-separation functionality and microalgal carbon fixation for sustainable biogas upgrading.

## Figures and Tables

**Table 2:** Water contact angle, surface free energy components, Zeta potential and adhesion free energy of the pristine and coated membranes and of *S. obliquus*.

Sample	θ_w_ (°)	γ_Tot_	γ_AB_	γ_LW_	γ_+_	γ_−_	ζ (mV)	ΔG_adh_	ΔG_adh,AB_	ΔG_adh,LW_
PES	75.7	38.41	4.70	33.71	0.63	8.78	−30.8	−41.74	−39.02	−2.73
PES-1.0%CS	89.2	32.93	1.62	31.31	0.32	2.05	8.1	−43.02	−40.8	−2.22
PES-1.5%CS	82.4	31.92	1.33	30.59	0.24	5.53	2.59	−43.77	−41.7	−2.07
PES-1.8%CS	86.4	32.79	1.04	31.75	0.39	2.67	4.8	−42.61	−40.29	−2.32
PES-2.0%CS	86.6	33.11	0.56	32.55	0.12	4.67	3.23	−45.47	−42.99	−2.48
PES-30%CS/IL	54.2	24.93	0.13	24.8	0.03	24.93	3.81	−47.06	−46.32	−0.75
PES-50%CS/IL	51.2	44.14	10.11	34.03	0.86	29.74	3.75	−47.51	−46.95	−0.56
PES-100%CS/IL	49.9	35.53	5.4	30.13	0.13	41.14	1.3	−45.74	−43.77	−1.97
*S. obliquus*	58.3	38.95	4.53	34.43	0.17	26.62	−39.73	—	—	—

Notes: θ_w_, water contact angle; γ_Tot_, γ_AB_, γ_LW_, total, acid–base and Lifshitz–van der Waals surface free energy; γ_+_, γ_−_, electron-acceptor and electron-donor components; ζ, Zeta potential; ΔG_adh_, adhesion free energy between membrane and *S. obliquus*. Surface free energies and ΔG_adh_ are in mJ·m^−2^. Values are single measurements. The coating was optimized on a porous PES model substrate.

**Table 3:** Techno-economic comparison of the microalgae membrane bioreactor with representative biogas upgrading technologies.

Technology	CH_4_ Purity	Advantages	Disadvantages	Cost (CNY·m^−3^·h^−1^)
Membrane separation	96%	Low operating cost; simple equipment and operation	Membrane prone to fouling; short lifetime; high cost	55,698
Water scrubbing	96%	Simple process; good regenerability; no chemical reagents	High operating cost and energy demand; large footprint; difficult CO_2_ recovery	74,984
Pressure swing adsorption	95%	Low energy demand; good regenerability; easy installation and start-up	High CH_4_ loss; high investment and operating cost; complex; prone to fouling	77,942
Cryogenic separation	97%	High CO_2_ separation; can produce liquid CH_4_	High energy; high investment and operating cost; complex equipment; high pressure and very low temperature required	—
Chemical absorption	96%	Fast process; small footprint; good regenerability	High investment cost; toxic absorbents; waste requires further treatment	70,540
Biological conversion	>98%	Low cost and energy; environmentally friendly	Rising pH and H_2_ partial pressure; requires nutrient addition; acid accumulation	—
MMBR (this work)	>96%	Low operating cost and energy; high mass-transfer efficiency; easy to regulate	Poorer regenerability; biofilm prone to detachment and ageing	—

Cost values for the conventional technologies are taken from Refs. [5,44] and are expressed per m^3^ h^−1^ of raw biogas treated. CNY, Chinese yuan. No cost value is given for the MMBR because the present system operates at laboratory scale and a verifiable cost analysis would require scale-up data; a techno-economic assessment of the MMBR is left for future work. CH_4_ purity values are representative figures reported for each technology.

## Data Availability

The data presented in this study are available on request from the corresponding author.

## Supplementary Materials

The following supporting information can be downloaded at: https://www.mdpi.com/article/10.3390/membranes16080279/s1. Text S1. Surface free energy and xDLVO interaction-energy equations; Figure S1: Characterization of the C_3_N_4_ and SC_3_N_4_ fillers; Figure S2: Characterization of the Pebax-based MMMs; Figure S3: Gas separation performance and stability of the Pebax-based MMMs; Figure S4: Supplementary characterization of the CA-based MMMs; Figure S5: FTIR of the coated PES membranes and N_2_ isotherms of the fillers; Figure S6: Surface and cross-sectional SEM of the CS/IL-coated PES membranes; Figure S7: Growth of attached microalgae; Figure S8: Full xDLVO profiles of the eight membranes; Figure S9: Seven-day adhesion time course; Figure S10: Biofilm morphology; Figure S11: Photograph of the laboratory-scale microalgae membrane bioreactor used in this study; Table S1: Codes and compositions of the membranes.
