# Supplementary material for: A Dual-Functional CO2-Selective Membrane for Biogas Upgrading in a Microalgae Membrane Bioreactor
Source: Membranes (Basel). 2026 Aug 21;16(8):279. doi: 10.3390/membranes16080279 (PMC13515271; doi:10.3390/membranes16080279)
Supplement: Supplementary file 1 [file membranes-16-00279-s001.zip › membranes-4504763-supplementary.pdf]

Supplementary Materials

Text S1. Surface free energy and xDLVO interaction-energy equations

When a microalgal cell approaches a membrane surface in water, three interfaces are involved (cell–membrane, membrane–liquid, and cell–liquid). The interfacial free energy of adhesion is

$$\Delta G_{adh} = \gamma_{sm} - \gamma_{sl} - \gamma_{ml} \quad \text{(S1)}$$

where γ_{sm}, γ_{sl}, and γ_{ml} are the interfacial free energies of the cell–membrane, membrane–liquid, and cell–liquid interfaces, respectively. Throughout, the subscripts m, s, and l denote the microalgal cell, the membrane (solid) surface, and the liquid, respectively. According to van Oss theory, the surface free energy comprises a non-polar Lifshitz–van der Waals (LW) component and a polar Lewis acid–base (AB) component, the latter split into an electron-acceptor (γ^+) and an electron-donor (γ^-) parameter:

$$\gamma = \gamma^{LW} + \gamma^{AB} = \gamma^{LW} + 2\sqrt{(\gamma^-\gamma^+)} \quad \text{(S2)}$$

The total adhesion free energy is the sum of the LW and AB contributions:

$$\Delta G_{adh} = \Delta G_{adh}^{LW} + \Delta G_{adh}^{AB} \quad \text{(S3)}$$

with

$$\Delta G_{adh}^{LW} = -2\,(\sqrt{(\gamma_m^{LW})} - \sqrt{(\gamma_l^{LW})})(\sqrt{(\gamma_s^{LW})} - \sqrt{(\gamma_l^{LW})}) \quad \text{(S4)}$$

$$\Delta G_{adh}^{AB} = 2[\sqrt{(\gamma_l^+)}(\sqrt{(\gamma_m^-)} + \sqrt{(\gamma_s^-)} - \sqrt{(\gamma_l^-)}) + \sqrt{(\gamma_l^-)}(\sqrt{(\gamma_m^+)} + \sqrt{(\gamma_s^+)} - \sqrt{(\gamma_l^+)}) - \sqrt{(\gamma_s^+\gamma_m^-)} - \sqrt{(\gamma_s^-\gamma_m^+)}] \quad \text{(S5)}$$

The three surface-tension parameters (γ^{LW}, γ^+, γ^-) were determined from the contact angles (θ) of three probe liquids (ultrapure water, diiodomethane, and ethylene glycol) using the Young equation:

$$\gamma_l(1 + \cos\theta) = 2\,(\sqrt{(\gamma_s^{LW}\gamma_l^{LW})} + \sqrt{(\gamma_s^+\gamma_l^-)} + \sqrt{(\gamma_s^-\gamma_l^+)}) \quad \text{(S6)}$$

The surface-tension components of the probe liquids were taken as constants (water, diiodomethane, and ethylene glycol; see the main text / thesis Table 2.4).

xDLVO interaction energy versus separation distance

According to the xDLVO theory, the total interaction energy between the two surfaces as a function of separation distance d comprises the LW, AB, and electric double-layer (EL) contributions:

$$\Delta G^{TOT}(d) = \Delta G^{LW}(d) + \Delta G^{AB}(d) + \Delta G^{EL}(d) \quad \text{(S7)}$$

The per-unit-area LW, AB, and EL interaction energies are

$$\Delta G^{LW}(d) = -(A/6)\,[a/d + a/(d+2a) + \ln(d/(d+2a))] \quad \text{(S8)}$$

where a is the microalgal cell radius (m), d is the separation distance (m), and A is the Hamaker constant, which characterizes the van der Waals interaction between the cell

and the membrane and is obtained from the non-polar (LW) adhesion free energy:

$$A = -12\pi\, d_0^2\, \Delta G_{adh}^{LW} \quad \text{(S9)}$$

with d_0 = 0.158 nm the minimum equilibrium separation between the cell and the membrane surface, and ΔG_{adh}^{LW} given by Eq. (S4). The acid–base interaction energy is

$$\Delta G^{AB}(d) = 2\pi\, a\, \lambda\, \Delta G_{adh}^{AB} \exp[(d_0 - d) / \lambda] \quad \text{(S10)}$$

where λ is the characteristic decay length of the liquid medium, taken as 0.6 nm (6 × 10^{-10} m), and ΔG_{adh}^{AB} is given by Eq. (S5). The electric double-layer interaction energy is

$$\Delta G^{EL}(d) = \pi \varepsilon_0 \varepsilon_r\, a\, (\zeta_m^2 + \zeta_s^2) \times \quad \text{(S11)}$$

$$[\, (2\zeta_m\zeta_s / (\zeta_m^2+\zeta_s^2)) \ln(\, (1 + \exp(-\kappa d)) / (1 - \exp(-\kappa d))\,) + \ln(1 - \exp(-2\kappa d))\,]$$

where ε_0 = 8.85 × 10^{-12} F m^{-1} is the vacuum permittivity, ε_r = 78.5 is the relative permittivity of the liquid, ζ_m and ζ_s are the Zeta potentials (V) of the microalgal cell and the membrane surface, a is the cell radius, and κ = 0.104 nm^{-1} is the inverse Debye length.

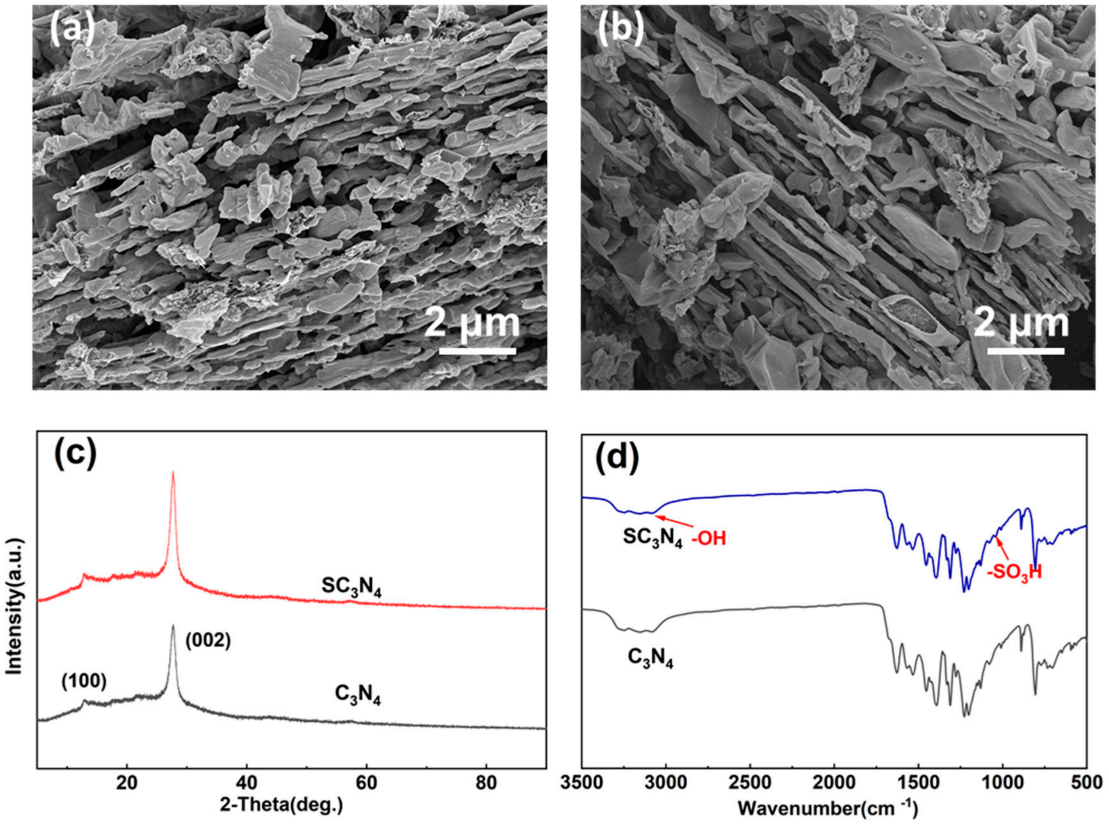

Figure S1. Characterization of the C_3N_4 and SC_3N_4 fillers. (a) SEM image of C_3N_4 and (b) SEM image of SC_3N_4; (c) XRD patterns and (d) FTIR spectra of C_3N_4 and SC_3N_4.

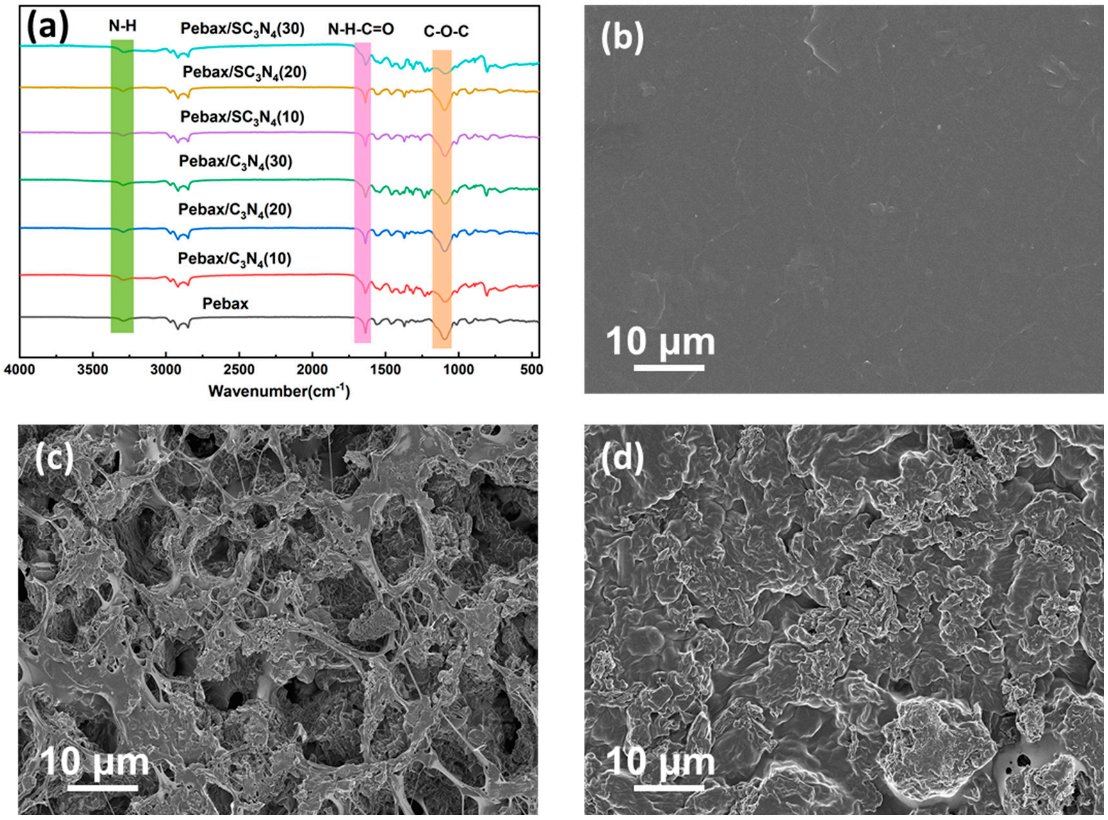

Figure S2. Characterization of the Pebax-based mixed-matrix membranes. (a) FTIR spectra of pristine Pebax and its C_3N_4- and SC_3N_4-loaded mixed-matrix membranes; surface SEM images of (b) pristine Pebax, (c) Pebax/C_3N_4(30) and (d) Pebax/SC_3N_4(30).

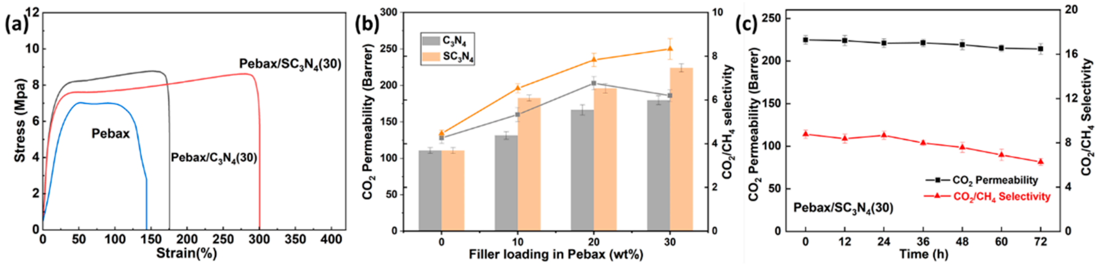

Figure S3. Gas separation performance and stability of the Pebax-based mixed-matrix membranes. (a) Mechanical properties of pristine Pebax and its mixed-matrix membranes; (b) effect of filler loading on CO_2 permeability and CO_2/CH_4 selectivity; (c) long-term operational stability of Pebax/SC_3N_4 (30) at 2 bar and 25 °C.

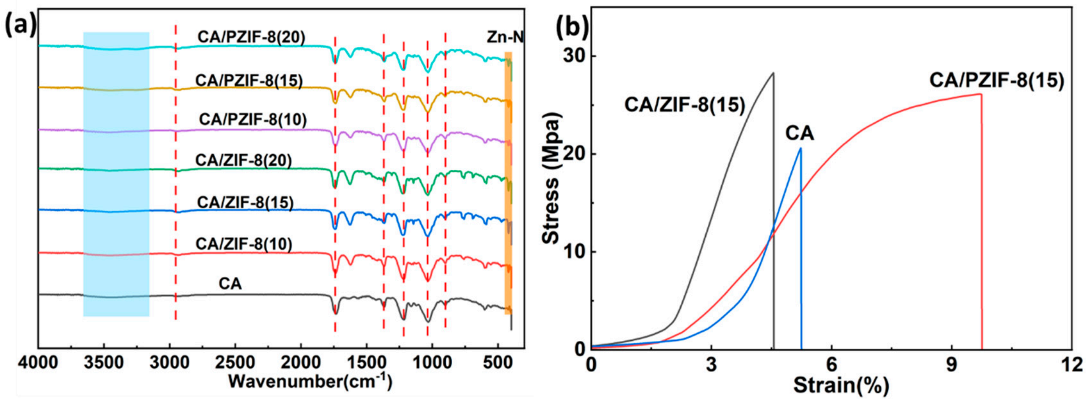

Figure S4. Supplementary characterization of the CA-based mixed-matrix membranes. (a) FTIR spectra of pristine CA and the CA/ZIF-8 and CA/PZIF-8 mixed-matrix membranes at different filler loadings; (b) mechanical properties of pristine CA and its mixed-matrix membranes (CA/PZIF-8(15): tensile strength 26.1 MPa, elongation at break 9.8%).

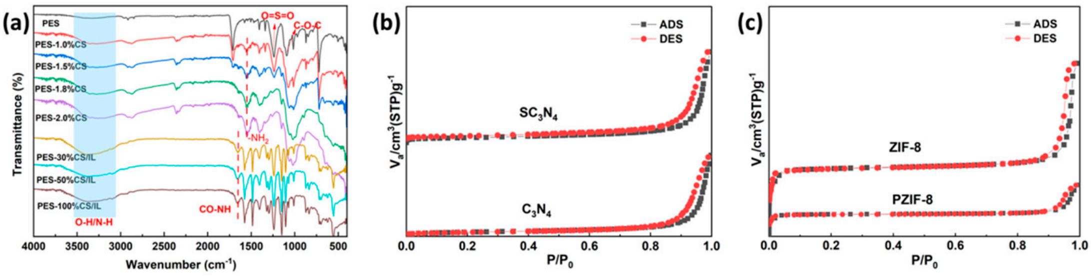

Figure S5. (a) FTIR spectra of the pristine and CS/IL-coated PES membranes; N_2 adsorption–desorption isotherms at 77 K of (b) C_3N_4 and SC_3N_4 and (c) ZIF-8 and PZIF-8.

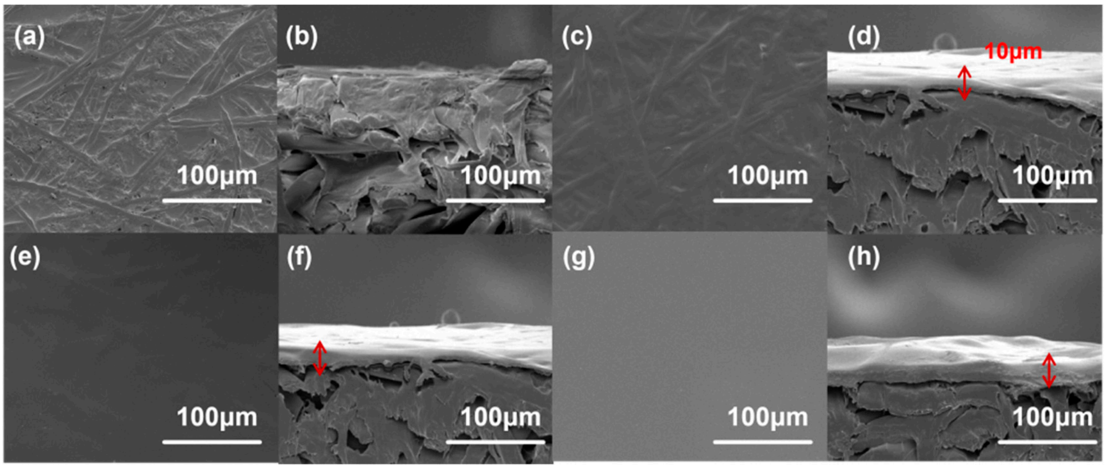

Figure S6. Surface and cross-sectional SEM images of the CS/IL-coated PES membranes: surface (a) and cross-section (b) of PES; surface (c) and cross-section (d) of PES-1.5%CS; surface (e) and cross-section (f) of PES-2.0%CS; surface (g) and cross-section (h) of PES-100%CS/IL.

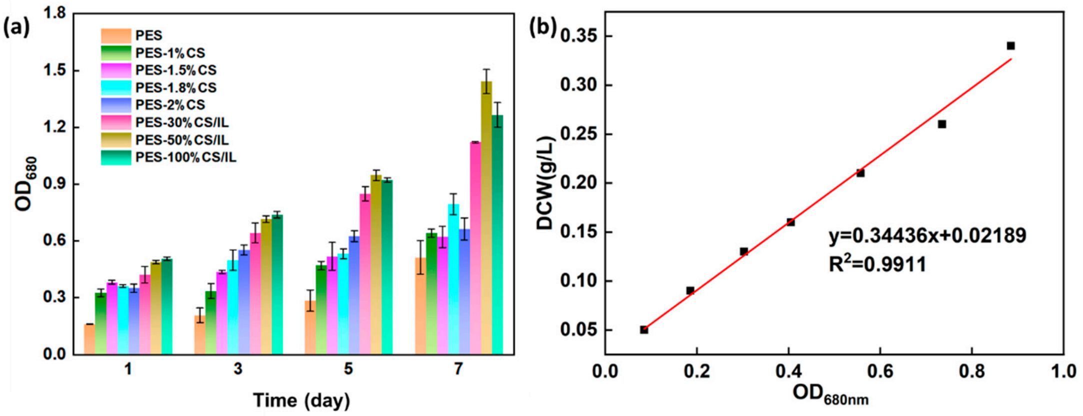

Figure S7. Growth of attached microalgae. (a) OD_{680} of *Scenedesmus obliquus* attached on the membranes at different culture stages; (b) Calibration curve between dry cell weight and OD_{680}.

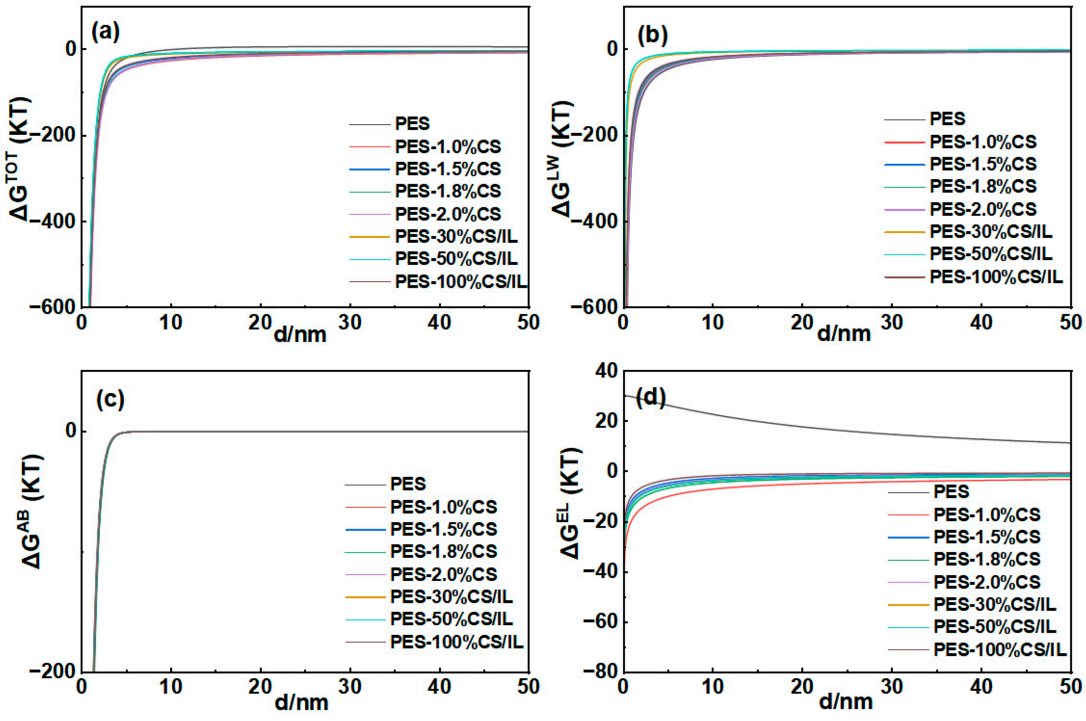

Figure S8. xDLVO interaction-energy profiles between the eight membranes and Scenedesmus obliquus as a function of separation distance: (a) total, (b) Lifshitz–van der Waals (LW), (c) acid–base (AB) and (d) electrostatic (EL) interaction energy.

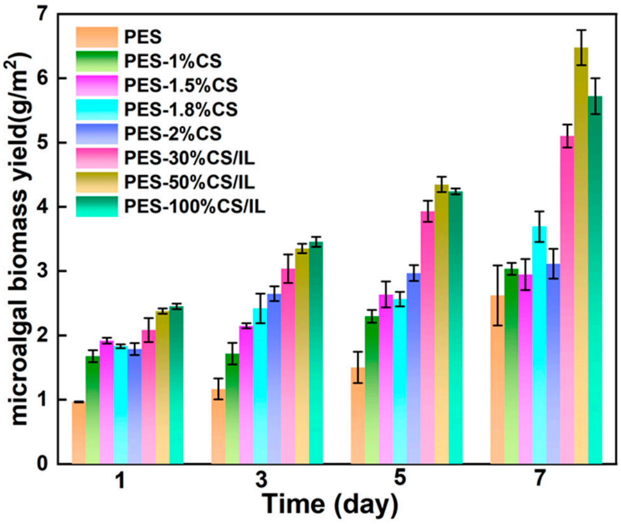

Figure S9. Accumulated microalgal adhesion on the membranes over seven days (mean ± standard deviation, n = 3).

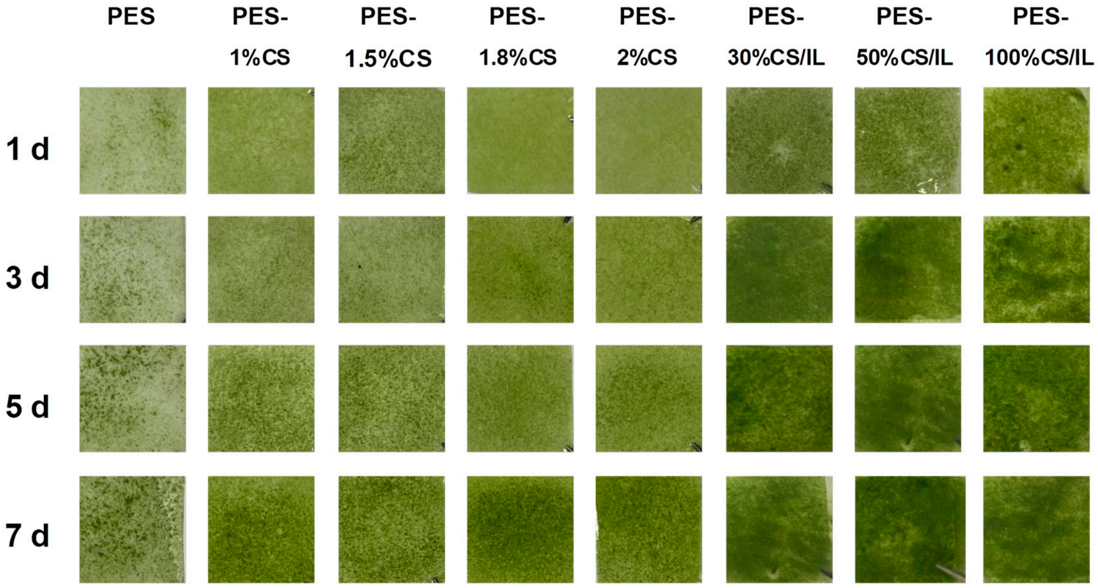

Figure S10. Morphology of the microalgal biofilm on the different membranes at different culture stages.

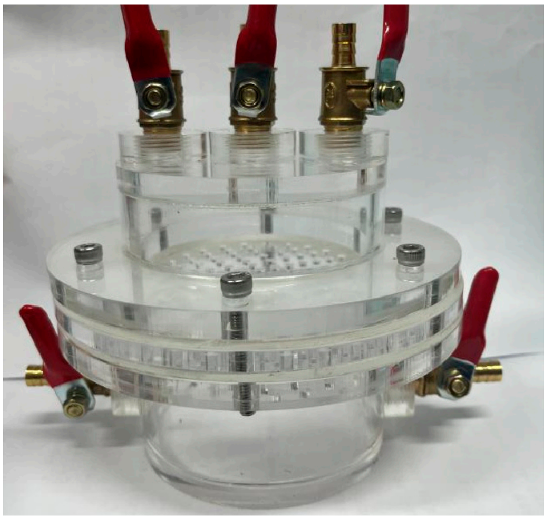

Figure S11. Photograph of the laboratory-scale microalgae membrane bioreactor used in this study.

Table S1. Codes and compositions of the membranes prepared in this study.

Sample code	Matrix	Filler / coating	Loading or ratio	Fabrication
PES	PES (commercial)	—	—	As received
PES-1.0%CS	PES	Chitosan (CS)	1.0 wt% CS solution	Spin-coating, vacuum-dried 12 h
PES-1.5%CS	PES	Chitosan (CS)	1.5 wt% CS solution	Spin-coating, vacuum-dried 12 h
PES-1.8%CS	PES	Chitosan (CS)	1.8 wt% CS solution	Spin-coating, vacuum-dried 12 h
PES-2.0%CS	PES	Chitosan (CS)	2.0 wt% CS solution	Spin-coating, vacuum-dried 12 h
PES-30%CS/IL	PES	CS grafted with [Emim][OAc] (CS/IL)	IL/CS = 30%	IL grafting then spin-coating
**PES-50%CS/IL**	**PES**	**CS/IL**	**IL/CS = 50%**	**IL grafting then spin-coating**
PES-100%CS/IL	PES	CS/IL	IL/CS = 100%	IL grafting then spin-coating
Pebax	Pebax	—	0	Solution casting, dried 40 °C 24 h
Pebax/C_3N_4(10)	Pebax	C_3N_4	10 wt%	Solution casting
Pebax/C_3N_4(20)	Pebax	C_3N_4	20 wt%	Solution casting
Pebax/C_3N_4(30)	Pebax	C_3N_4	30 wt%	Solution casting
Pebax/SC_3N_4(10)	Pebax	SC_3N_4(sulfonated C_3N_4)	10 wt%	Solution casting
Pebax/SC_3N_4(20)	Pebax	SC_3N_4	20 wt%	Solution casting
Pebax/SC_3N_4(30)	Pebax	SC_3N_4	30 wt%	Solution casting
CA	CA	—	0	Solution casting
CA/ZIF-8(10)	CA	ZIF-8	10 wt%	Solution casting
CA/ZIF-8(15)	CA	ZIF-8	15 wt%	Solution casting
CA/ZIF-8(20)	CA	ZIF-8	20 wt%	Solution casting
CA/PZIF-8(10)	CA	PZIF-8 (PEI-grafted ZIF-8)	10 wt%	Solution casting
**CA/PZIF-8(15)**	**CA**	**PZIF-8**	**15 wt%**	**Solution casting**
CA/PZIF-8(20)	CA	PZIF-8	20 wt%	Solution casting
**IL-CS@CA/PZIF-8(15)**	**CA/PZIF-8(15)**	**CS/IL surface coating**	**IL/CS = 50%**	**Coat CS/IL on CA/PZIF-8(15), 60 °C 24 h**

Notes：CS, chitosan; IL, ionic liquid ([Emim][OAc], 1-ethyl-3-methylimidazolium acetate); CS/IL, IL-grafted chitosan; SC_3N_4, sulfonated g-C_3N_4; PZIF-8, polyethyleneimine-grafted ZIF-8. For the CS series the number denotes the chitosan solution concentration (wt%); for the CS/IL series it denotes the ionic-liquid-to-chitosan mass ratio; for the mixed-matrix membranes it denotes the filler loading (wt%), defined as $m_{filler}/(m_{filler}+m_{polymer})$. The PES series was used as a model substrate.
